# Effects of Fatigue on Six-Degree-of-Freedom Knee Kinematics in Military Personnel Marching on a Treadmill

**DOI:** 10.3390/bioengineering13101142

**Published:** 2026-09-30

**Authors:** Longxiang Li, Longzhou Hua, Mingwei Liu, Ye Luo, Yingjie Lyu, Fei Tian, Shaobai Wang

**Affiliations:** 1 School of Exercise and Health, Shanghai University of Sport, Shanghai 200438, China; 2 Department of Rehabilitation Medicine, Changzhi Medical College, Changzhi 046000, China

**Keywords:** knee joint, six degrees of freedom, gait analysis, exercise fatigue, military training

## Abstract

**Background**: Knee injuries are common during military training, and fatigue alters lower-limb movement patterns, increasing injury risk. However, little is known about fatigue effects on knee kinematics during military marching. Conventional gait analysis is limited to sagittal plane motion, leaving multi-planar knee function poorly understood. **Objective**: This study investigated six-degree-of-freedom (6DOF) knee kinematic changes in healthy male military personnel marching at 8 km/h on a treadmill before and after fatigue, focusing on initial contact (IC), stance-phase flexion peak (SPF), and stance-phase extension peak (SPE). **Methods**: Thirty-eight healthy male soldiers marched on a treadmill at 8 km/h. A motion capture system (infrared stereo cameras + reflective markers) collected 6DOF kinematic data. Fatigue was induced by loaded marching (15 kg vest), with the endpoint defined as Borg RPE ≥ 18 or heart rate ≥ 90% of age-predicted maximum. Post-fatigue data were collected at the same speed. Flexion, rotation, and anterior–posterior displacement at IC, SPF, and SPE were compared pre- vs. post-fatigue. The Holm–Bonferroni correction was applied for multiple comparisons. **Results**: After Holm–Bonferroni correction, knee flexion angles decreased significantly at SPF (3.64°, *p* = 0.009, Cohen’s d = 0.52) and SPE (2.75°, *p* = 0.032, d = 0.41), with a non-significant trend at IC (2.53°, *p* = 0.086). Internal rotation shifted toward external rotation at IC (2.48°, *p* = 0.039, d = 0.46) and SPE (2.99°, *p* = 0.036, d = 0.54), with a non-significant trend at SPF (*p* = 0.076). No other kinematic changes, including anteroposterior displacement, exceeded the system’s measurement error threshold or remained significant after correction. **Conclusions**: Fatigue reduces knee flexion during the stance phase at 8 km/h (with the most robust changes at SPF and SPE) and shifts internal–external rotation toward external rotation at IC and SPE. These 6DOF kinematic changes may compromise knee stability and increase ACL and patellofemoral injury risk.

## 1. Introduction

Military training is characterized by high-intensity, repetitive, and complex environments, resulting in a significantly higher incidence of lower-limb musculoskeletal injuries among military personnel than in the general population [1,2]. As the primary weight-bearing joint, the knee is subjected to complex loads during marching, running, sudden stops, and weight-bearing exercises, and its biomechanical changes are a key factor in common training injuries, including ACL tears and patellofemoral pain syndrome [3]. Fatigue is an inevitable physiological condition in military training that impairs neuromuscular control [4], proprioception, and joint stability, thereby altering movement patterns and increasing injury risk [5]. Therefore, understanding fatigue-induced changes in knee kinematics is critical for optimizing training and preventing injuries.

Gait analysis is widely used to quantify knee kinematics. Conventional approaches emphasize sagittal plane motion, often neglecting frontal and transverse plane kinematics [6]. However, the knee is a six-degree-of-freedom (6DOF) joint, comprising three translational (anterior–posterior, medial–lateral, vertical) and three rotational (flexion–extension, adduction–abduction, internal–external) components. Sagittal-only analysis cannot fully capture knee stability under dynamic loading, missing critical frontal and transverse plane changes linked to non-contact knee injuries [7,8]. Recent advances have made 6DOF kinematic analysis a frontier in gait research [6], with applications including load-altered knee kinematics [9], ACL injury assessment [10], and speed-dependent gait patterns [11]. The portable OptiKnee system enables real-time 6DOF kinematic measurement with high accuracy and clinical usability [12], and has been widely adopted in sports biomechanics [13].

However, most fatigue-gait studies focus on single-plane or gross gait parameters [14], with limited attention to multi-planar knee kinematics pre- and post-fatigue. The IC, SPF, and SPE phases are critical high-load periods [15], and their kinematic changes may directly reflect fatigue effects on joint stability. Yet, few studies have quantitatively compared knee kinematics across these phases, and even fewer have examined fatigue-induced gait changes in military personnel at 8 km/h on a treadmill—a speed near the energy-optimal transition between walking and running (approx. 7–8 km/h) [16].

This study therefore examined 6DOF knee kinematic changes in 38 healthy male military personnel marching at 8 km/h on a treadmill, pre- and post-fatigue, focusing on IC, SPF, and SPE, to provide biomechanical evidence for injury prevention, fatigue management, and rehabilitation. We hypothesized that fatigue would reduce knee flexion during stance and shift rotation toward external rotation.

## 2. Materials and Methods

### 2.1. Participants

Thirty-eight healthy male active-duty soldiers were recruited through an internal military announcement (convenience sampling). Their mean age was 20.8 ± 1.4 years, and mean BMI was 23.5 ± 2.0 kg/m^2^. All participants met the following inclusion criteria: (1) no lower-limb musculoskeletal injury within the past 6 months (confirmed by orthopedic evaluation, including negative Lachman and pivot shift tests); (2) no cardiovascular or metabolic diseases (confirmed by resting ECG and normal blood pressure); (3) completion of at least three marching drills per week (≥3 km each, verified by training logs). All participants were tested on the right knee joint.

The study protocol was approved by the Institutional Review Board (IRB) of Shanghai University of Sport (Approval No.: 102772023RT134, Approval Date: 13 October 2023). All participants provided written informed consent prior to participation.

A companion study based on a partially overlapping cohort has been accepted for publication in the Chinese journal *Journal of Medical Biomechanics* [17]. That paper reports gait kinematics at 3 km/h in 48 soldiers, of whom 20 also participated in the present 8 km/h study. The two studies focus on different walking speeds and report different kinematic parameters. The 8 km/h data presented here have not been published elsewhere.

### 2.2. Instrumentation

(1) A motion capture system (OptiKnee, Innomotion, Shanghai, China) was used to collect three-dimensional knee motion data. The system includes: (a) four infrared reflective markers (OK-Marquer, Innomotion) attached to the subject’s lower limbs; (b) a dual-lens infrared stereo camera (60 Hz) tracking marker trajectories; (c) a handheld probe for identifying the knee joint center and bony landmarks [18]; and (d) companion software (Optimum, version 3.0, Innomotion, Shanghai, China) that automatically detects gait cycles and computes three-dimensional kinematic parameters. The system uses anatomical landmark calibration and a rigid-body assumption to minimize skin motion artifact, and its validation studies reported translational and rotational errors of <1.3 mm and <0.9°, respectively [12,19].

(2) A treadmill (Innomotion, Shanghai, China) was used to control marching speed, with a range of 0–10 km/h and a stability error of ±0.1 km/h.

#### Calculation of 6DOF Knee Kinematics

The 6DOF knee kinematics were calculated using the OptiKnee system. Anatomical coordinate systems for the femur and tibia were established according to the ISB recommendations [17]. The relative motion of the femur with respect to the tibia was represented by a homogeneous transformation matrix:

pFT=RFT⋅PFF+tFT

where pFT is the position vector of a point in the femoral coordinate system expressed in the tibial coordinate system,RFT is the rotation matrix from the femoral to the tibial coordinate system, and tFT the translation vector from the femoral origin to the tibial origin, expressed in the tibial coordinate system. The three components of tFT in the tibial coordinate system correspond directly to the three translational degrees of freedom: anterior–posterior translation, medial–lateral translation, and proximal–distal translation. The three rotational degrees of freedom were extracted from RFT as Cardan angles using an X–Y–Z sequence, where the X-axis corresponds to flexion–extension, the Y-axis to internal–external rotation, and the Z-axis to abduction–adduction. This approach allows for a clear separation of translations and rotations: the translation vector tFT describes the position of the femoral origin relative to the tibial origin, while the rotation matrix RFT describes the orientation of the femur relative to the tibia. The accuracy and repeatability of this method have been previously validated, with translational and rotational errors of <1.3 mm and <0.9°, respectively [19].

### 2.3. Experimental Protocol

#### 2.3.1. Pre-Experiment Preparation

Participants completed a 5–10 min warm-up (light jogging and dynamic lower-body stretches) and received instructions on the procedure and marching guidelines.

#### 2.3.2. Pre-Fatigue Data Collection

**Figure 1:**
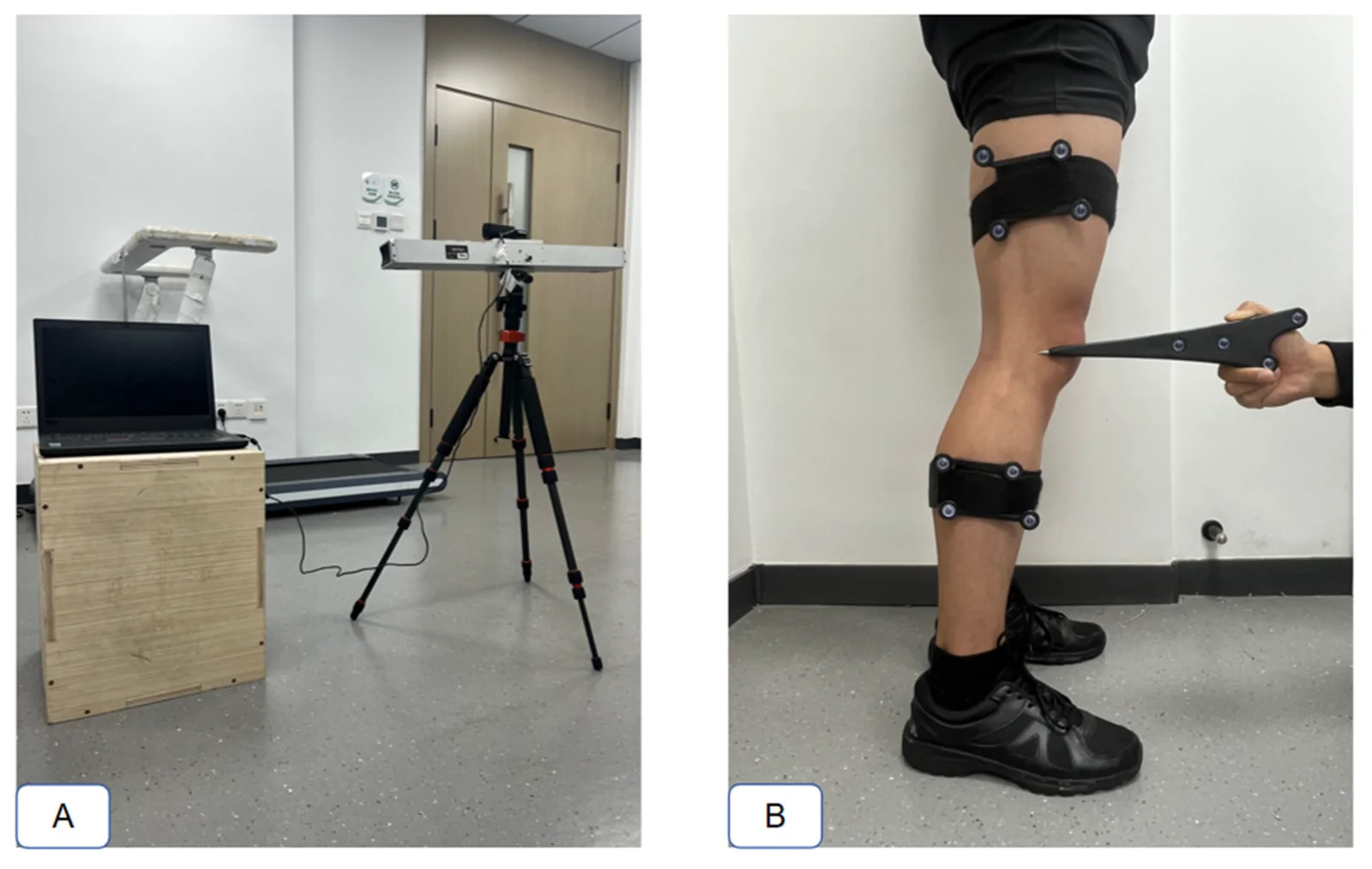
Instrument setup and three-dimensional spatial positioning of the right knee joint. (**A**) Instrument setup; (**B**) Probe directed toward the lateral femoral condyle.

Reflective markers were attached 8 cm above the lateral femoral condyle and 3 cm below the tibial plateau (Figure 1), following the OptiKnee manual to ensure placement over bony landmarks. After confirming secure attachment and no interference with knee movement, participants marched on the treadmill at 8 km/h. Once gait stabilized (~3 min), 15 s of data were recorded to obtain at least 10 complete gait cycles per participant.

#### 2.3.3. Exercise-Induced Fatigue

A standardized fatigue protocol was used: participants marched on a level treadmill [20] at 8 km/h while wearing a 15 kg weighted vest [21]. Fatigue endpoint was defined as any of: (a) Borg RPE [22] ≥ 18 (6–20 scale), a criterion used in prior military fatigue studies [23]; (b) heart rate ≥ 90% of age-predicted maximum (220 − age), monitored every 10 s via telemetry; (c) involuntary reduction in marching speed > 0.5 km/h, simulating battlefield speed maintenance requirements.

#### 2.3.4. Post-Fatigue Data Collection

Immediately after reaching the fatigue endpoint, participants removed the weighted vest and marched again at 8 km/h. Once gait stabilized, data were collected using the same criteria (15 s, ≥10 cycles) to ensure testing under fatigued conditions.

#### 2.3.5. Gait Cycle Definition and Parameter Extraction

A total of 10 consecutive complete gait cycles were selected from the stable gait recording for each participant. Cycles with marker dropout, incomplete marker trajectories, or obvious gait instability were excluded from analysis. If any of the initially selected cycles were invalid, they were replaced by the next consecutive valid cycles until 10 valid cycles were obtained for each participant. Kinematic parameters were averaged within each participant to obtain a single representative value, and these individual averages were used for statistical analysis (n = 38). Each cycle was defined by heel strike, normalized to 0–100%, and divided into stance and swing phases [24]. At 8 km/h, the stance phase comprised ~40% of the gait cycle [25], representing the primary weight-bearing and loading phase. Average motion curves pre- and post-fatigue were compared to observe overall kinematic trends. Kinematic parameters at IC, SPF, and SPE were extracted from each participant’s individual gait curves and compared to quantify fatigue effects on knee function.

### 2.4. Statistical Analysis

SPSS 27.0 was used for data analysis. Data are presented as mean ± SD. Normality was assessed using the Shapiro–Wilk test. Paired *t*-tests were used for normally distributed data; otherwise, Wilcoxon signed-rank tests were applied. Given the multiple comparisons performed across three gait events and three kinematic parameters, the Holm–Bonferroni sequential correction was applied to control the family-wise error rate. Effect sizes (Cohen’s d) were calculated for significant findings to assess the magnitude of differences. Statistical significance was set at α = 0.05 (two-tailed).

## 3. Results

### 3.1. Characteristics of the Participants

All 38 participants completed the study with no dropouts or data anomalies, and all data were complete and valid. Table 1 summarizes participant characteristics (age, height, weight, and BMI).

### 3.2. Changes in Three-Dimensional Kinematic Parameters of the Knee Joint

**Figure 2:**
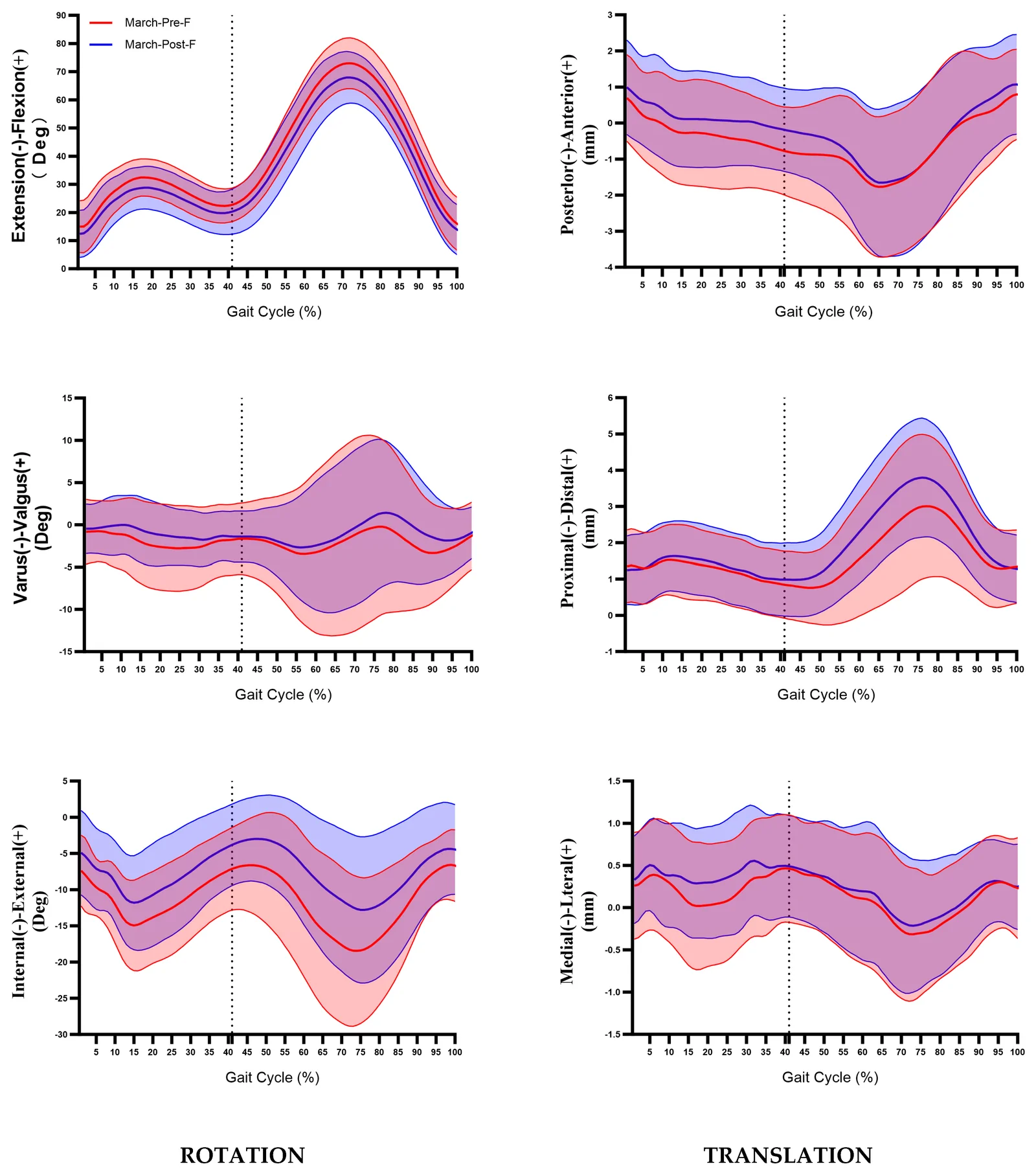
Three-dimensional knee kinematic curves before and after fatigue. The gait cycle was normalized from heel strike to the next heel strike on the same foot (0–100%). Solid lines represent the mean pre-fatigue and post-fatigue values; shaded areas indicate ±1 standard deviation (SD) across participants. March-Pre-F and March-Post-F denote pre- and post-fatigue mean curves.

Figure 2 shows 6DOF knee kinematic curves during gait before and after fatigue at 8 km/h. Post-fatigue, knee flexion angle decreased throughout stance, with internal-external rotation shifting toward external rotation. These changes were most pronounced at IC, SPF, and SPE.

Table 2 compares pre- and post-fatigue knee kinematics. Knee flexion angle decreased significantly at IC (2.53°, 95% CI: −4.98 to −0.08, *p* = 0.043), SPF (3.64°, 95% CI: −5.96 to −1.32, *p* = 0.003), and SPE (2.75°, 95% CI: −4.98 to −0.52, *p* = 0.016), indicating reduced shock absorption. Internal rotation angle decreased (shifted toward external rotation) at IC (2.48°, 95% CI: 0.53 to 4.43, *p* = 0.013), SPF (3.08°, 95% CI: 0.17 to 5.99, *p* = 0.038), and SPE (2.99°, 95% CI: 0.53 to 5.45, *p* = 0.018). No other kinematic parameters, including anteroposterior and mediolateral translations, exhibited changes exceeding the system’s measurement error threshold (<1.3 mm for translations) or remained statistically significant after Holm–Bonferroni correction.

## 4. Discussion

This study analyzed 6DOF knee kinematic changes in healthy male military personnel marching at 8 km/h on a treadmill before and after fatigue, focusing on IC, SPF, and SPE. Post-fatigue, knee flexion angle decreased significantly at SPF and SPE (with a trend at IC), and internal rotation shifted toward external rotation at IC and SPE. These findings provide quantitative evidence for understanding reduced knee dynamic stability under fatigue and offer a biomechanical perspective for injury prevention in military training.

From a biomechanical perspective, the observed reduction in knee flexion during stance may alter the distribution of joint contact forces. A more extended knee shifts the ground reaction force vector anteriorly, increasing the external flexion moment that the quadriceps must resist. This may elevate patellofemoral joint stress and could increase anterior tibial shear force via the quadriceps–ACL interaction mechanism. Similarly, the shift toward external rotation may change tibiofemoral contact mechanics and alter load distribution across the menisci. However, these interpretations are based on previously established biomechanical models and should be considered hypothesis-generating rather than definitive.

Knee flexion angles at IC, SPF, and SPE decreased following fatigue, with reductions of 2.53°, 3.64°, and 2.75°, respectively. After Holm–Bonferroni correction, the changes at SPF (*p* = 0.009, Cohen’s d = 0.52) and SPE (*p* = 0.032, d = 0.41) remained significant, while the IC change did not survive correction (*p* = 0.086). Although a 3.64° reduction in knee flexion was statistically significant, its biomechanical meaningfulness should be interpreted with caution. Computational simulations have suggested that a 3–5° reduction in early-stance knee flexion may increase ACL loading by approximately 10–15% under identical vertical ground reaction forces. However, no universally accepted minimal clinically important difference (MCID) exists for gait kinematics, and the cumulative effect of such small changes over thousands of cycles remains to be established. This “straightening” may represent a compensatory mechanism to reduce muscle work and maintain marching speed [4]. A straighter knee absorbs ground reaction forces less effectively, transmitting forces more directly to bones and ligaments, which may be associated with increased ACL and articular surface load [3]. Reduced knee flexion angle is a known predictor of non-contact ACL injuries [6], and computer simulations suggest that insufficient flexion may increase anterior tibial shear and knee valgus moment, potentially elevating ACL loading [8]. Reduced flexion may also alter patellar tracking, potentially affecting patellofemoral joint pressure [3]. The observed reduction in knee flexion during stance may also be interpreted from an energy-cost perspective. At 8 km/h, which approximates the walk–run transition speed where metabolic cost is minimized [16], fatigue likely compels the neuromuscular system to adopt a more extended knee posture to reduce the demand on knee flexors—particularly the hamstrings and gastrocnemius—which are heavily recruited during the deceleration and early loading phases of stance [4]. This energy-sparing strategy, however, comes at a biomechanical cost. A more extended knee shifts the ground reaction force vector anterior to the knee joint center, increasing the external knee flexion moment that must be resisted primarily by the quadriceps [3]. This quadriceps-dominant response not only may elevate patellofemoral joint stress through altered patellar tracking [3], but may also increase anterior tibial shear force via the quadriceps–ACL interaction mechanism, wherein strong quadriceps contraction at low flexion angles generates substantial anterior translation of the tibia [26]. While these load increases may be tolerable in isolated steps, their cumulative effect over thousands of gait cycles during prolonged or repetitive military training could contribute to the progressive microdamage of ligamentous and articular tissues. This mechanistic pathway is consistent with the established epidemiological link between high-volume marching and the elevated incidence of ACL and patellofemoral injuries in military populations [1,2].

In addition to sagittal plane changes, fatigue also significantly affected the knee joint movement pattern in the transverse plane. The present study observed significant changes in internal–external rotation angles during the IC and SPE phases (with a non-significant trend at SPF after correction), specifically a marked decrease in internal rotation angle (i.e., a shift toward relative external rotation), with an average change ranging from 2.48° to 3.08°. Since internal rotation was defined as negative and external rotation as positive, a decrease in internal rotation angle indicates a tendency toward relative external rotation after fatigue. This finding is consistent with Thomas et al. (2010), who found that combined hamstring and quadriceps fatigue significantly increased knee external rotation angle at initial contact and at peak ground reaction force [27]. Murdock and Hubley-Kozey (2012) also confirmed that high-intensity quadriceps fatigue significantly increased knee external rotation during walking in healthy young adults [28]. The trends observed in this study align with these previous findings, all supporting that fatigue can alter the transverse plane movement pattern of the knee joint.

Beyond the neuromuscular accounts proposed in previous studies [29,30], the observed shift toward relative external rotation may also be explained by mechanical coupling with the concurrently reduced knee flexion angle. In a more extended knee position, the tibiofemoral articular geometry is substantially altered: the femoral condyles roll posteriorly on the tibial plateau, and the contact interface presents a flatter profile to the femur. This geometric change reduces the articular constraint that normally drives the “screw-home” mechanism—a phenomenon in which the tibia undergoes obligatory internal rotation during the final degrees of knee extension as a result of the asymmetric geometry of the medial and lateral tibial plateaus [31,32]. With reduced flexion, this rotational locking mechanism becomes less effective, allowing greater rotational laxity and a consequent shift in the tibia toward a relatively external resting position [32]. This interpretation is supported by cadaveric studies demonstrating that tibial rotation varies systematically as a function of knee flexion angle [31]. Importantly, because this explanation derives directly from our measured kinematic data rather than from unmeasured muscle activation patterns, it remains within the evidential scope of this study. That said, the relative contributions of passive articular geometry versus active neuromuscular control to the observed rotational changes cannot be disentangled from the present data and warrant future investigation using integrated motion capture and electromyography.

During the stance phase of gait, appropriate axial rotation of the knee helps distribute torsional loads; however, excessive or abnormal internal/external rotation may alter patellofemoral alignment, potentially contributing to patellofemoral pain syndrome [3]. Moreover, reduced ability to control rotation may decrease the knee’s capacity to counteract ground-reaction torques, potentially increasing shear stress on the menisci and ligaments. Netravali et al. found that abnormal external rotation of the tibia relative to the femur (approximately 3.2°) alters tibiofemoral contact mechanics and is associated with degenerative changes in articular cartilage [33]. These results suggest that changes in transverse plane movement patterns after fatigue should be a key focus in gait assessment and injury prevention.

Regarding translational kinematics, no significant fatigue-induced changes were observed in anteroposterior or mediolateral translations at any of the three gait events examined. The only nominally significant change in anteroposterior displacement at SPE (0.57 mm) fell below the system’s measurement error threshold (<1.3 mm) [19], and did not survive the Holm–Bonferroni correction; therefore, it was not interpreted as a definitive biomechanical adaptation.

Notably, no significant fatigue-induced changes were observed in adduction–abduction angles or medial–lateral translation across any of the three gait events examined. This suggests that frontal-plane knee control may be relatively preserved at the moderate marching speed of 8 km/h, even under the fatigue state induced by our protocol. This finding aligns with previous observations that frontal-plane knee mechanics are more sensitive to variations in running speed and to unanticipated cutting or landing maneuvers than to fatigue alone [11,14]. It is also possible that the relatively low-impact nature of treadmill marching at 8 km/h—as opposed to overground running or jump-landing tasks—did not sufficiently stress the frontal-plane stabilizers (e.g., gluteus medius and the lateral collateral ligament complex) to provoke detectable changes. Alternatively, the absence of significant findings in the frontal plane may reflect limited statistical power following the Holm–Bonferroni correction for multiple comparisons, particularly if the true effect size in this plane is small. Future studies incorporating higher-demand tasks—such as overground marching with load at speeds exceeding 10 km/h, or maneuvers involving sudden directional changes—are warranted to determine whether frontal-plane alterations emerge under more challenging conditions.

Traditional sagittal-plane analysis alone is insufficient for assessing fatigue-related injury risk. The portable, non-invasive OptiKnee system (translation error < 1.3 mm, rotation error < 0.9°) enables rapid gait assessment in field settings and could support dynamic monitoring to identify individuals at high fatigue risk and guide personalized training load adjustments. The 6DOF kinematic changes identified in this study—particularly the reduced flexion and altered rotational patterns during stance—may serve as quantitative biomechanical markers for monitoring knee joint status during military training, potentially enabling early detection of at-risk individuals before injuries occur. From a practical standpoint, the 6DOF kinematic metrics identified in this study may serve as quantitative indicators for monitoring knee joint status during military training. Unit trainers and physical therapists could use portable motion analysis systems, such as the OptiKnee system, to periodically assess knee kinematics during treadmill marching at 8 km/h. A reduction in knee flexion or a shift toward external rotation beyond the system’s measurement error may signal fatigue-related changes in joint stability, prompting adjustments in training load, marching speed, or rest schedules. In rehabilitation settings, these metrics could help track recovery progress and guide return-to-duty decisions by identifying residual kinematic deficits. Although further validation is needed, incorporating such assessments into routine training monitoring may help reduce the cumulative risk of ACL and patellofemoral injuries associated with repetitive, high-volume marching.

This study has several limitations. First, regarding study design, the absence of a non-fatigued control group means that physiological fatigue adaptations cannot be completely separated from time-dependent behavioral drift in the treadmill environment; therefore, it cannot be entirely ruled out that some of the small kinematic changes observed may have been partly influenced by behavioral drift rather than fatigue alone. In addition, only one marching speed (8 km/h) was tested, and the fatigue protocol combined subjective and heart-rate criteria, so central and peripheral fatigue effects could not be distinguished. Second, regarding measurement and analysis, this study measured kinematics only and did not collect EMG or ground reaction forces, so joint loading and injury outcomes were not directly assessed; skin-mounted markers are also susceptible to soft tissue artifact, particularly for small transverse rotations and translations, and residual artifact cannot be completely excluded. Third, regarding sample characteristics, all participants were healthy young males, limiting generalizability, and the sample size, although adequate for detecting differences in flexion and rotation, may have been insufficient for detecting smaller effect sizes in the frontal plane. The small AP displacement change fell below the measurement error threshold and was not interpreted as a meaningful biomechanical adaptation. Future work should integrate surface EMG and force plates to establish a coupled kinematic–kinetic–EMG model and conduct prospective injury surveillance to clarify causal relationships between fatigue-induced gait changes and injury occurrence.

## Figures and Tables

**Table 1:** Characteristics of the included participants.

Variable	Values (Mean ± SD)
Age, y	20.79 ± 1.42
Height, cm	177.18 ± 4.88
Body mass, kg	73.91 ± 7.29
BMI	23.54 ± 2.05

Values are expressed as mean ± SD unless otherwise indicated.

**Table 2:** A comparison of 3D knee kinematics during gait events while marching: before and after fatigue.

	Variable	Marching		
		Pre	Post	*p*
IC	Flexion angle (°)	14.95 (9.23)	12.42 (8.40)	0.043 *
	Internal–external (°)	−7.36 (4.91)	−4.88 (5.85)	0.013 *
SPF	Flexion angle (°)	33.22 (6.63)	29.58 (7.47)	0.003 *
	Internal–external (°)	−14.53 (6.26)	−11.45 (6.83)	0.038 *
SPE	Flexion angle (°)	21.77 (6.01)	19.02 (7.40)	0.016 *
	Internal–external (°)	−7.21 (5.82)	−4.22 (5.56)	0.018 *

Notes: Data are presented as mean (standard deviation). IC = initial contact; SPF = stance phase flexion peak; SPE = stance phase extension peak. Positive values for flexion angle indicate flexion; for internal-external rotation, positive = external rotation, negative = internal rotation. * *p* < 0.05 indicates a statistically significant difference (paired *t*-test, uncorrected). After Holm–Bonferroni correction, significant differences remained for flexion at SPF and SPE, and for internal-external rotation at IC and SPE; all other comparisons were non-significant.

## Data Availability

The datasets generated and analyzed during the current study are not publicly available due to confidentiality restrictions for military personnel. The statistical analyses were performed using standard procedures in SPSS 27.0; no custom analysis code was developed. De-identified data may be available from the corresponding author upon reasonable request and with appropriate institutional approval.
